# Population Genetics of *Sillago japonica* Among Five Populations Based on Mitochondrial Genome Sequences

**DOI:** 10.3390/genes16080978

**Published:** 2025-08-20

**Authors:** Beiyan Zhu, Tianxiang Gao, Yinquan Qu, Xiumei Zhang

**Affiliations:** Fishery College, Zhejiang Ocean University, Zhoushan 316022, China; ziky9911@163.com (B.Z.); qyquan@njfu.edu.cn (Y.Q.)

**Keywords:** *Sillago japonica*, SNPs, mitochondrial genome, genetic differentiation, genetic diversity

## Abstract

Objectives: *Sillago japonica* is a commercially important marine fish species in the Northwestern Pacific, and understanding its genetic diversity and population structure is crucial for germplasm resource conservation and elucidating population evolution mechanisms. This study specifically aimed to systematically explore the genetic diversity and population structure of *S. japonica* across five geographic regions (DJW, YSW, ST, ZS, and RS) in its distribution range. Methods: A total of 50 *S. japonica* individuals from the five geographic regions were analyzed using high-throughput mitochondrial genome sequencing data. We identified single nucleotide polymorphisms (SNPs) and insertion-deletion (InDel) loci, followed by comprehensive population genetic analyses including phylogenetic tree construction, principal component analysis (PCA), ADMIXTURE analysis, and calculation of genetic differentiation indices (*Fst*) and genetic diversity parameters. Results: A total of 2966 SNPs and 414 insertion-deletion loci were identified. Phylogenetic tree topology, PCA, and ADMIXTURE 1.3.0 analysis consistently showed low genetic differentiation among the five populations, a pattern supported by low pairwise *Fst* values ranging from 0.00047 to 0.05589, indicating extensive gene flow across regions. Genetic diversity parameters varied slightly among populations: observed heterozygosity (0.00001–0.00528), expected heterozygosity (0.04552–0.07311), percentages of polymorphic loci (19.41–30.36%), and nucleotide diversity (0.04792–0.07697). Conclusions: The low genetic differentiation and diversity observed in *S. japonica* populations may result from the combined effects of historical bottleneck-induced gene pool reduction and extensive gene flow. These findings provide essential theoretical support for formulating targeted conservation strategies for *S. japonica* germplasm resources and further studies on its population evolution mechanisms.

## 1. Introduction

*Sillago japonica* (Japanese whiting), a member of the genus *Sillago*, belongs to the family Sillaginidae within the order Perciformes. It is a commercially valuable offshore fish in the Northwestern Pacific [1,2]. Widely distributed along the coasts of China, Korea, and Japan, it primarily inhabits sandy substrates in shallow coastal waters and estuarine environments [3,4]. Due to its rapid growth and high culinary value, *S. japonica* plays a crucial role in fisheries production and marine ecosystem dynamics [5,6]. In recent years, *S. japonica* has faced growing threats from anthropogenic and environmental stressors. Studies have found significant declines in catch yields of *S. japonica* across its key distribution areas in the Northwestern Pacific, with overfishing and coastal habitat degradation identified as primary drivers [7].

The mitochondrial genome, an essential constituent of the eukaryotic genome, represents a discrete genetic entity in eukaryotic cells that is independent of the nuclear genome and encodes genes vital for energy metabolism and other critical cellular functions [8]. The mitochondrial genome, characterized by maternal inheritance, structural stability, moderate evolutionary rate, and absence of recombination, serves as an ideal molecular marker for studying population genetic structure [9,10,11]. Mitochondrial single-nucleotide polymorphisms (SNPs) are particularly valuable due to their high mutation rate, which enables fine-scale resolution of population differentiation and recent demographic events [12]. Compared with traditional mitochondrial markers (e.g., COI or D-loop), genome-wide mitochondrial SNPs provide a higher resolution for detecting subtle genetic structures and historical migration patterns [13]. In the field of ichthyological research, mitochondrial genome analysis has emerged as a pivotal approach for exploring the population genetic structure, genetic diversity, and phylogenetic relationships of fishes [14,15]. By decoding mitochondrial genome sequences, researchers are able to trace the historical dynamics of fish populations and reveal the genetic connectivity among different geographic populations, providing a theoretical basis for the sustainable management of fishery resources [16,17]. Despite these developments, research on the genetic status of *S. japonica* remains limited. For instance, traditional morphological surveys and low-resolution molecular marker methods such as microsatellites fail to detect fine-scale genetic changes [2]. Notably, declines in genetic diversity among marine fish have been widely documented. For example, large yellow croaker (*Larimichthys crocea*) has witnessed a sharp decline in both population and genetic diversity due to overfishing and habitat degradation. The once-abundant wild resources have been severely depleted, and the genetic variation within the species has been greatly reduced, which has affected its adaptability and evolutionary potential [18]. Given the documented population declines and preliminary signs of genetic erosion in *S. japonica* due to anthropogenic and environmental stressors [19,20], alongside the broader disruption of marine habitats by ocean warming, pollution, and overfishing [21,22,23], there is an urgent need for advanced genetic techniques to clarify its genetic structure. High-resolution tools such as mitochondrial genome analysis combined with mtSNP screening can provide the detailed genetic insights required for effective management.

Hence, we employed mitochondrial genome analysis to investigate *S. japonica* from five geographic populations (Zhoushan, Shantou, Rushan, Ise Bay, and Tokyo Bay). Based on mitochondrial genomes and population genetic analysis, this study aims to elucidate the genetic structure and genetic diversity of the species, providing critical insights for the sustainable management and conservation of *S. japonica* resources.

## 2. Materials and Methods

### 2.1. Sample Collection and DNA Extraction

**Figure 1:**
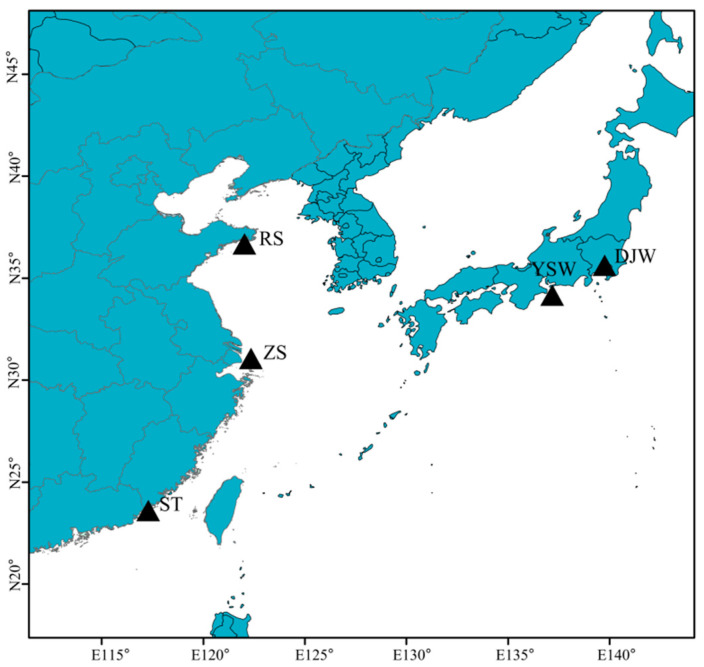
Sample collection locations of *Sillago japonica*. Capital letters mean the abbreviations of sampling locations.

Fifty samples of *S. japonica* were collected from five distinct geographical populations located in ZS, ST, RS, YSW, and DJW (sampling locations shown in Figure 1). Dorsal muscle tissues were immediately immersed in liquid nitrogen and stored at −80 °C until further analysis. Total DNA was extracted from muscle tissues using the standard phenol–chloroform method [24]. DNA concentration and purity (OD260/OD280 ratio) were measured with a NanoDrop 2000C spectrophotometer (Thermo Fisher Scientific, Waltham, MA, USA), while integrity was verified by 1.0% agarose gel electrophoresis. Qualified DNA samples were dissolved in sterilized ddH_2_O for subsequent experiments.

### 2.2. Library Construction, Illumina Sequencing, and Mitochondrial Genome Assembly

Genomic DNA extracted from 50 *S. japonica* samples was digested using Hae III and EcoR I restriction enzymes. Subsequently, P1 adapters with barcodes and sequencing primers were ligated to the digested products, followed by the ligation of P2 adapters to the fragmented DNA. Adapter-ligated sequences were amplified via PCR, and the products were purified using the AxyPrep DNA Gel Extraction Kit (AxyGEN, Union City, CA, USA) and adjusted to a concentration of 1 ng/μL. The insert sizes of the libraries were evaluated using an Agilent 2100 bioanalyzer (Agilent Technologies, Santa Clara, CA, USA). Libraries with effective concentrations greater than 2 nM, as determined by quantitative qPCR, underwent paired-end sequencing (2 × 100 bp) on the Illumina HiSeqTM2500 platform.

Raw sequencing reads were filtered using FastQC v0.11.5 to remove sequences with more than 10% low-quality bases (Q-score < 20), lengths shorter than 50 bp, or residual adapters [25]. The filtered reads were then aligned to the mitochondrial genome reference sequence (accession number: AP006803.1) using BWA v0.7.17 (Burrows–Wheeler Aligner) software with the settings “mem-t 4-k 32-M” [26]. The alignment reads in SAM format were extracted, converted into BAM format, and sorted according to genomic positions by SAMtools v1.9 with a maximum of 1000 reads per position in each BAM file [27]. Next, the mitochondrial genome was assembled de novo using NOVOPlasty v4.2 [28], with the reference sequence serving as a seed (K-mer = 33; insert size = 300 bp) to initiate the assembly process. The assembly parameters were configured to prioritize accurate circular genome reconstruction. Finally, the assembled mitochondrial genome was annotated using the MitoFish v4.09 online platform.

### 2.3. Variant Calling, Population Structure, Diversity, and Divergence

Variant calling was performed using GATK v.4.0.3.0 according to the recommended best practices workflow [29]. First, GATK HaplotypeCaller was employed to identify general variants for each individual sample. Subsequently, the GenotypeGVCFs function was utilized to merge these individual variant calls into a single comprehensive variant calling file. This two-step procedure was implemented to enhance the accuracy of variant identification, involving re-genotyping and quality recalibration processes on the combined VCF file. SNPs were detected from the alignment of all Illumina short reads using samtools and bcftools with default settings. Rigorous filtering criteria were applied to these SNPs: (1) SNPs detected exclusively in either the SAMtools/BCFtools or GATK; (2) SNPs with a read depth exceeding 1000 or below 5; (3) non-biallelic SNPs; (4) SNPs with a missing data rate higher than 40%; (5) SNPs in repeat regions; (6) SNPs within 5 base pairs of adjacent variant sites.

To infer phylogenetic relationships, a gene tree was constructed using SNPs from single-copy gene regions. Two popular phylogenetic software packages, RAxML v8.2.11 [30] with the GTRCAT model and IQ-Tree v1.6.12 [31] with the automatically estimated optimal substitution model, were utilized to generate maximum-likelihood (ML) trees. PCA was conducted using GCTA software v1.94.1 [32] to explore genetic variation among samples. The ancestral population structure of the five *S. japonica* populations was analyzed using ADMIXTURE software v.1.3.0, testing different ancestral population sizes (K = 2–7). Admixture analysis was performed with parameter standard errors estimated via 200 bootstrap replicates. Population genetic parameters, including expected heterozygosity (*H*e), observed heterozygosity (*H*o), percentages of polymorphic loci (PPB), nucleotide diversity (*π*), and pairwise (*Fst*) were computed for each SNP using the population analysis module in Stacks [33].

### 2.4. Selection Pressure Analysis

We performed sliding-window-based *Fst* analysis to detect potential positive selection signals. We calculated pairwise *Fst* values among five geographic populations with a non-overlapping 500 bp sliding window using VCFtools v0.1.17 [34]. Windows in the top 5% of *Fst* values were considered candidate selection regions.

## 3. Results

### 3.1. Mitochondrial Genome Assembly and Genetic Variant Mining

To systematically investigate the population structure and genetic diversity of *S. japonica*, this study conducted complete genome sequencing using high-throughput sequencing technology on 50 individuals collected from five geographic populations (10 specimens per population): Zhoushan (ZS), Shantou (ST), Rushan (RS), Ise Bay (YSW), and Tokyo Bay (DJW) (Figure 1).

**Figure 2:**
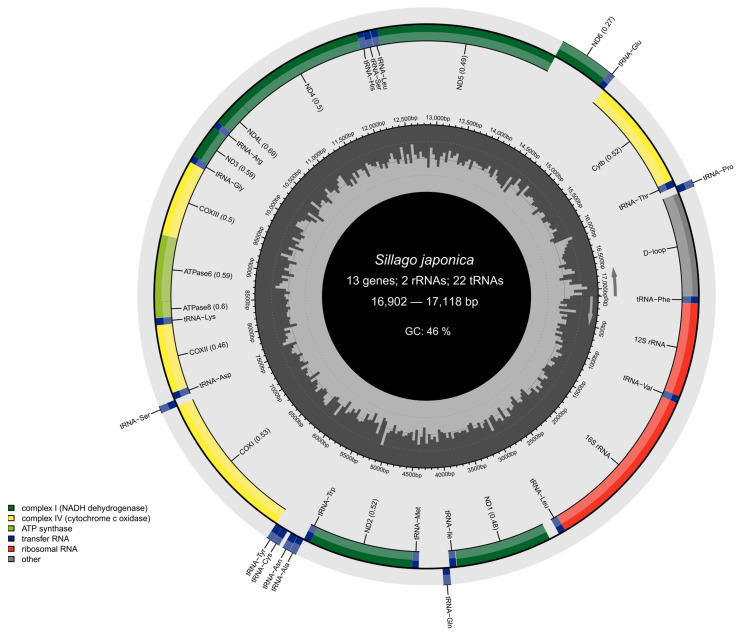
The mitochondrial annotation circular map of *S. japonica*.

The assembled mitochondrial DNA (mtDNA) of *S. japonica* exhibited a typical closed circular double-stranded structure, consistent with that of most fish mtDNAs (Figure 2). The sequence lengths ranged from 16,902 to 17,118 base pairs, with an average GC content of 46%. All sequenced mitochondrial genomes comprised the canonical set of 13 protein-coding genes, 22 transfer RNA (tRNA) genes, 2 ribosomal RNA (rRNA) genes, and a noncoding control region (D-loop).

A total of 2966 single-nucleotide polymorphisms (SNPs) and 414 insertion–deletion (Indels) variants were identified in 50 individuals of *S. japonica*. The results showed the presence of 88 synonymous variants. Additionally, 1458 variants were identified in the upstream regulatory regions, and 1650 variants were detected in the downstream regulatory regions. In exon regions, 148 variants were identified, with intergenic variants being the least frequent (10 variants). Intragenic variants accounted for 106, whereas frameshift variants represented the rarest category, comprising only 5 (Table 1).

### 3.2. Population Structure of S. japonica

**Figure 3:**
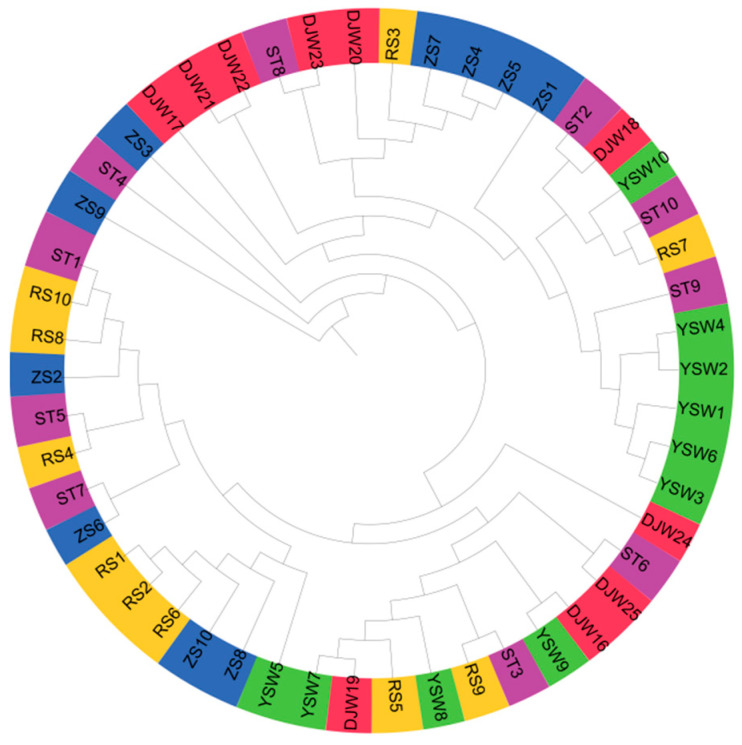
Phylogenetic relationship of 50 *S. japonica* individuals.

A phylogeny of *S. japonica* from five populations revealed minimal population divergence (Figure 3). All 50 individuals were randomly distributed across the tree, showing no geographically correlated clustering patterns. These findings indicated the absence of significant genetic divergence among the five populations despite geographical isolation, suggesting low genetic differentiation levels and extensive inter-population gene flow.

**Figure 4:**
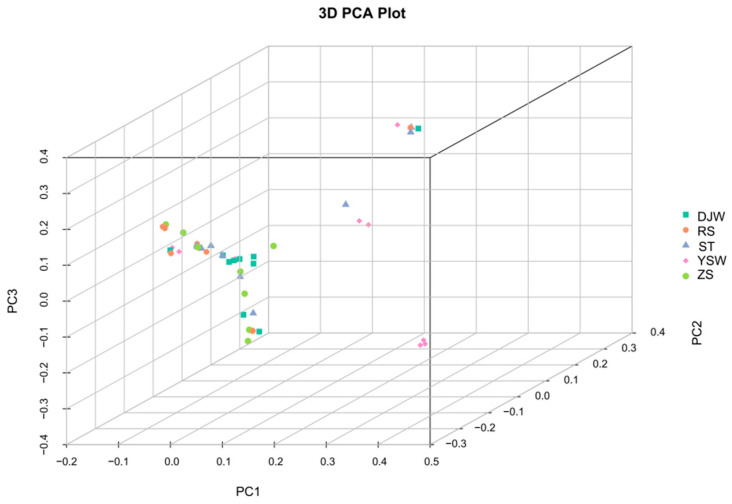
PCA of the five *S. japonica* populations.

Individuals of *S. japonica* exhibited overlapping distributions in the PC1-PC3 space, precluding the formation of discrete clusters based on geographic origin (Figure 4). The three principal components (PC1, PC2, and PC3) collectively failed to discriminate among the five populations. These results from the PCA corresponded closely with those of the phylogenetic analysis, providing additional evidence for the lack of distinct genetic structuring related to geographical location.

**Figure 5:**
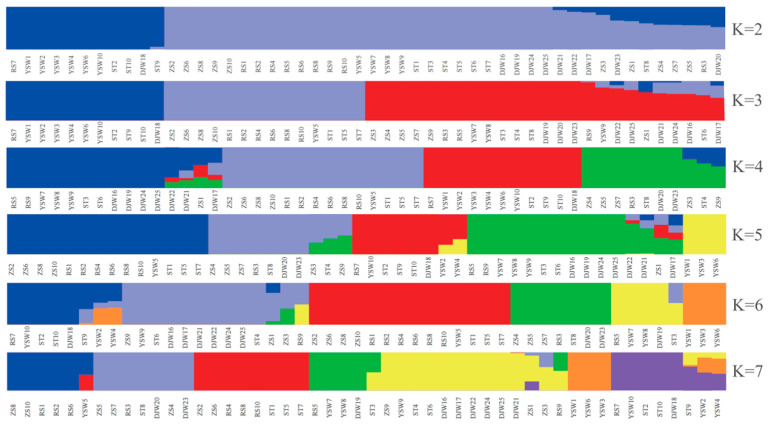
ADMIXTURE plot for *S. japonica* showing the distribution of K = 2–7 genetic clusters.

**Figure 6:**
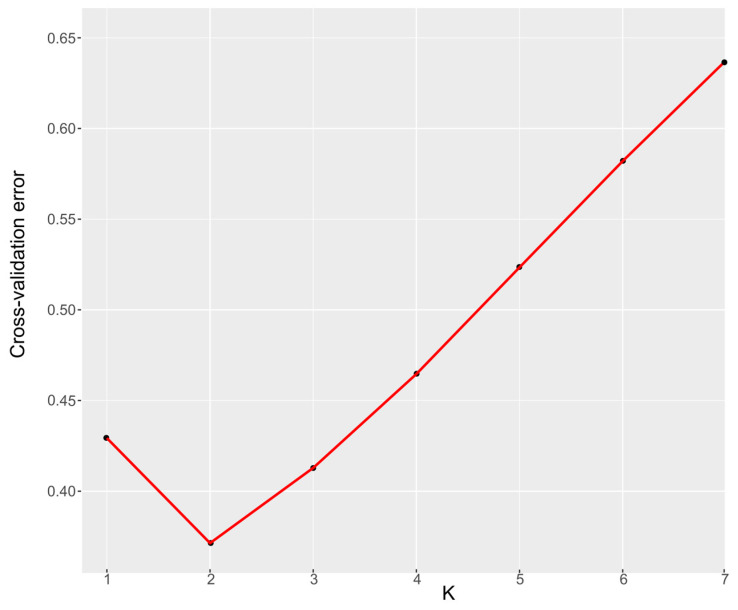
Cross-validation plot displaying CV-error versus K, suggests K = 2 is the best fit.

The ancestral population structure among five *S. japonica* geographical populations was estimated from population sizes K = 2–7 by ADMIXTURE analyses (Figure 5). The population size (K = 2) with the smallest cross-validation error (Figure 6) was determined and supported the phylogenetic topology and PCA. A phylogeny of these *S. japonica* individuals partitioned these samples into two distinct groups. The 11 samples were clustered in the first group and most belonged to the YSW populations. The remaining 39 samples from five populations were represented in the second group. The structure among five populations showed no obvious genetic differentiation among different geographical populations, indicating gene flow among these populations.

### 3.3. Genetic Diversity and Divergence of S. japonica Population

**Figure 7:**
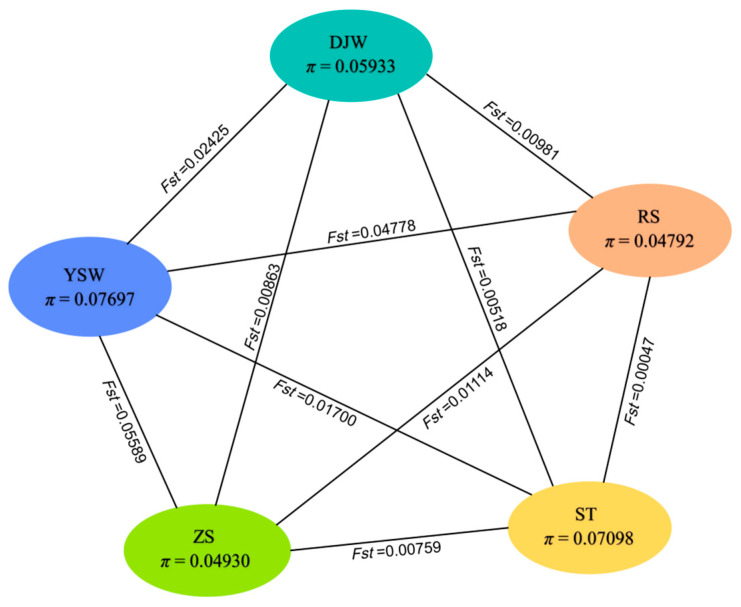
Summary of nucleotide diversity (*π*) and population divergence (*Fst*) in five geographical populations. Values in parentheses represent measures of nucleotide diversity of each group, and values between pairs indicate population divergence.

The analyzed populations (DJW, RS, ST, YSW, and ZS) exhibited low genetic diversity, as evidenced by the following indexes (Table 2). Observed heterozygosity (*H*o) approached zero across all populations (range: 0.00001–0.00528), while expected heterozygosity (*H*e) remained low (0.04552–0.07311), indicating severely depleted genetic variation. The percentage of polymorphic loci (PPB%) ranged narrowly between 19.41% and 30.36%, further confirming limited genetic variability. Among the populations, YSW and ST displayed marginally higher diversity (*H*e = 0.07311 and 0.06743, respectively; PPB% ≈ 30%), whereas RS and ZS showed the lowest variability (*H*e ≤ 0.04683; PPB% < 20%). The slightly elevated *H*o in ZS (0.00528) may indicate recent gene flow or differential selection pressures. Additionally, the YSW population displayed the highest nucleotide diversity (*π*) at 0.07697, reflecting the greatest degree of nucleotide sequence variation among these populations (Figure 7). These patterns collectively point to significant genetic erosion, likely resulting from historical bottlenecks, prolonged isolation, and/or intensive artificial selection in these populations.

In addition, we observed that the pairwise population divergence (*Fst*) values among populations ranged from 0.00047 to 0.05589, reflecting a genetic differentiation pattern among the five *S japonica* populations. Among them, the *Fst* between the YSW and ZS populations reached 0.05589, whereas the *Fst* between the RS and ST populations was only 0.00047, indicating minimal genetic differentiation and relatively frequent gene flow between them.

### 3.4. Genome-Wide Selection Pressure Analysis

**Figure 8:**
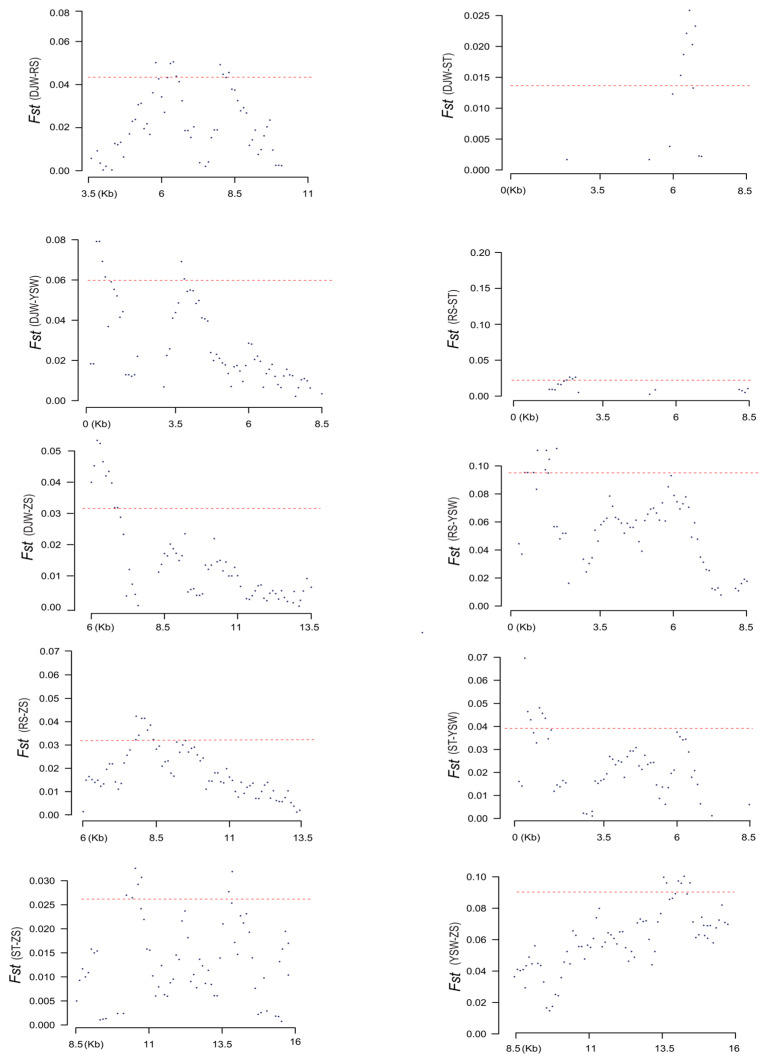
Population divergence (top 5% *Fst*) among DJW, YSW, ZS, RS, and ST. Each dot on the mitochondrial genome represents the *Fst* value at a specific position, reflecting the level of genetic differentiation in that region; the red line serves as the screening threshold, and the dots above the red line are exactly the 5% of highly differentiated loci under selection pressure in the mitochondrial genome.

Through comprehensive mitochondrial genome scanning, selection pressure signatures in *S. japonica* were uncovered. The study identified 5% of loci under selection across the genome, with particularly pronounced selection signals detected in certain regions (Figure S1 and Figure 8). The results visually illustrate the *Fst* values among five populations, providing insights into their genetic differentiation and the distribution of selective sweep regions across the genome. While the highest *Fst* value was indicative of significant genetic differentiation, observed specifically between the YSW and ZS populations, it is important to note that selection pressures were not limited to this pair but were widespread across the populations studied. These findings collectively suggest that *S. japonica* has undergone complex selective pressures, which have contributed to its genetic diversity and adaptability. Moreover, the selected regions exhibited minimal overlap between populations. For instance, DJW vs. RS displayed a divergent peak at 3.5–11 Kb (*Fst* = 0.043), whereas DJW vs. YSW showed a distinct peak at 0–5 Kb (*Fst* = 0.060). The results indicated that distinct selective pressures likely drive their genetic differentiation. These insights underscore the necessity for a detailed genetic understanding of *S. japonica* to guide effective conservation and management strategies amid escalating environmental pressures.

## 4. Discussion

In the present study, we investigated the genetic population structure of *S*. *japonica* populations in the Northwestern Pacific using mitochondrial genome data. Phylogenetic tree topology, PCA, and ADMIXTURE analyses consistently demonstrated a lack of significant genetic differentiation among the five *S. japonica* populations. In addition, pairwise *Fst* values among *S. japonica* populations ranged from 0.00047 to 0.05589, significantly lower than those reported for numerous other marine species. For example, *Esox americanus vermiculatus* exhibited *Fst* values of 0.205 to 0.480 [35], and *Dendropoma lebeche* showed an *Fst* of 0.410 [36]. Collectively, these results indicate that *S. japonica* populations display low genetic differentiation, suggesting extensive gene flow across their geographic distribution.

The finding aligns with previous studies on marine fish species with high dispersal potential, where larval mobility or adult migratory behavior facilitates genetic homogenization despite spatial separation [37,38]. For *S. japonica*, its life history traits, such as pelagic larval stages and occupation of contiguous coastal habitats, may promote connectivity among populations. However, the absence of geographic clustering contrasts with reports on similar samples [39], where ocean currents and biogeographic barriers drive population structuring. This discrepancy may reflect differences in molecular markers. Our results share certain similarities with prior mitochondrial SNP-based investigations of marine fish population structures. For instance, studies on Atlantic salmon have confirmed that mitochondrial SNPs exhibit stable polymorphism, efficiently reflect historical population dynamics, reduce biases in genetic analyses, and serve as reliable tools for analyzing genetic patterns of populations across broad geographical ranges [40]. The population-specific mitochondrial SNPs identified in *Megalobrama terminalis* from the Heilong River echo the conclusion of this study that “life history traits affect population connectivity,” indicating that mitochondrial SNPs can effectively capture adaptive genetic signals of species to their habitats [41]. This discrepancy highlights a critical methodological insight: mitochondrial genomes, while valuable for phylogenetic studies, may fail to capture recent or subtle genetic differentiation due to their conserved nature and slow mutation rate. The matrilineal inheritance of mtDNA further amplifies stochastic lineage sorting, potentially masking population subdivisions detectable through biparentally inherited nuclear markers [42,43]. For example, the absence of geographic clustering in our data contrasts with nuclear marker-based studies of the same species [39], where oceanographic barriers drove subtle population structuring. This discrepancy underscores the need for complementary nuclear genome analyses (e.g., RAD-seq or whole-genome sequencing) to assess contemporary gene flow and localized adaptation. Future studies integrating mitochondrial and nuclear data could resolve whether the observed panmixia reflects true demographic connectivity or masks incipient divergence driven by selective pressures (e.g., temperature or salinity gradients) [39].

Genetic diversity plays a crucial role in the survival, reproduction, fitness, and evolution of species worldwide [44]. Frequently, metrics including *H*o, *H*e, PPB, and π calculated from single-nucleotide polymorphisms serve as key indicators for assessing population genetic diversity [45]. In our study, the low observed heterozygosity (*H*o) across all populations further supports limited genetic divergence, potentially indicating recent population expansions or bottlenecks. Notably, the ZS population exhibited marginally higher *H*o than others, possibly due to its location in a hydrodynamically complex region (Zhoushan Archipelago), which may harbor microhabitats buffering against genetic drift. Conversely, the YSW population’s elevated nucleotide diversity (*π*) suggests historical stability or admixture events.

The overall low genetic diversity observed in *S. japonica* populations raises concerns about their adaptive capacity amid escalating environmental stressors. Anthropogenic pressures, such as overfishing and habitat degradation, have been linked to reduced genetic diversity in marine fishes [46,47], and our findings align with declining trends reported for Northwest Pacific *S. japonica* stocks [48]. The predominance of regulatory region variants (upstream/downstream SNPs) over coding regions implies that non-neutral evolutionary forces, such as purifying selection, may constrain mitochondrial protein evolution, a pattern consistent with maternal inheritance and functional constraints.

*S. japonica* populations overall display low genetic differentiation, suggesting extensive gene flow across their geographic distribution. Nevertheless, subtle genetic variations were observed among populations. The YSW and ZS populations showed the highest pairwise *Fst* (0.05589), though still below the threshold for significant differentiation (*Fst* > 0.15) [49]. While this may suggest limited gene flow between these groups, the mitochondrial data alone cannot determine whether this reflects neutral processes (e.g., drift) or incipient ecological divergence. Therefore, precautionary conservation measures, including minimizing anthropogenic habitat fragmentation, could be considered to maintain potential connectivity. For the RS and ST populations with an *Fst* of 0.00047, the genetic homogeneity implies high contemporary gene flow, supporting unified management of their shared habitat.

## 5. Conclusions

In summary, our study demonstrated that *S. japonica* has undergone complex selective pressures contributing the low levels of genetic differentiation and genetic diversity among YSW, DJW, ZS, RS, and ST populations. These findings informed conservation and breeding strategies, as selecting genetically distant populations for propagation can enhance genetic exchange, expand the gene pool, and improve adaptability to environmental changes and long-term survival. Overall, our study provides a theoretical foundation for future research on *S. japonica* conservation and sustainable resource utilization.

## Figures and Tables

**Table 1:** Summary of the SNPs and Indels identified in five populations of *Sillago japonica.*

Category	*S. japonica* Populations
SNPs	2966
Indels	414
Synonymous variants	88
Upstream	1458
Downstream	1650
Exon	148
Intergenic variants	10
Intragenic variants	106
Frameshift variants	5
Noncoding transcript exon variants	33

**Table 2:** Population genetic diversity parameters of *S. japonica.*

Population	Observed Heterozygosity (*H*o)	Expected Heterozygosity (*H*e)	Percentages of Polymorphic Loci (PPB) %
DJW	0.00019	0.05636	26.54360
RS	0.00006	0.04552	19.85996
ST	0.00001	0.06743	30.36283
YSW	0.00001	0.07311	28.38956
ZS	0.00528	0.04683	19.41439

## Supplementary Materials

The following supporting information can be downloaded at https://www.mdpi.com/article/10.3390/genes16080978/s1, Figure S1: Population divergence (*Fst*) between the DJW, YSW, ZS, RS, and ST.
