# Supplementary material for: Population Genetics of Sillago japonica Among Five Populations Based on Mitochondrial Genome Sequences
Source: Genes (Basel). 2025 Aug 20;16(8):978. doi: 10.3390/genes16080978 (PMC12385479; doi:10.3390/genes16080978)
Supplement: Supplementary file 1 [file genes-16-00978-s001.zip › genes-3773279-supplementary.pdf]

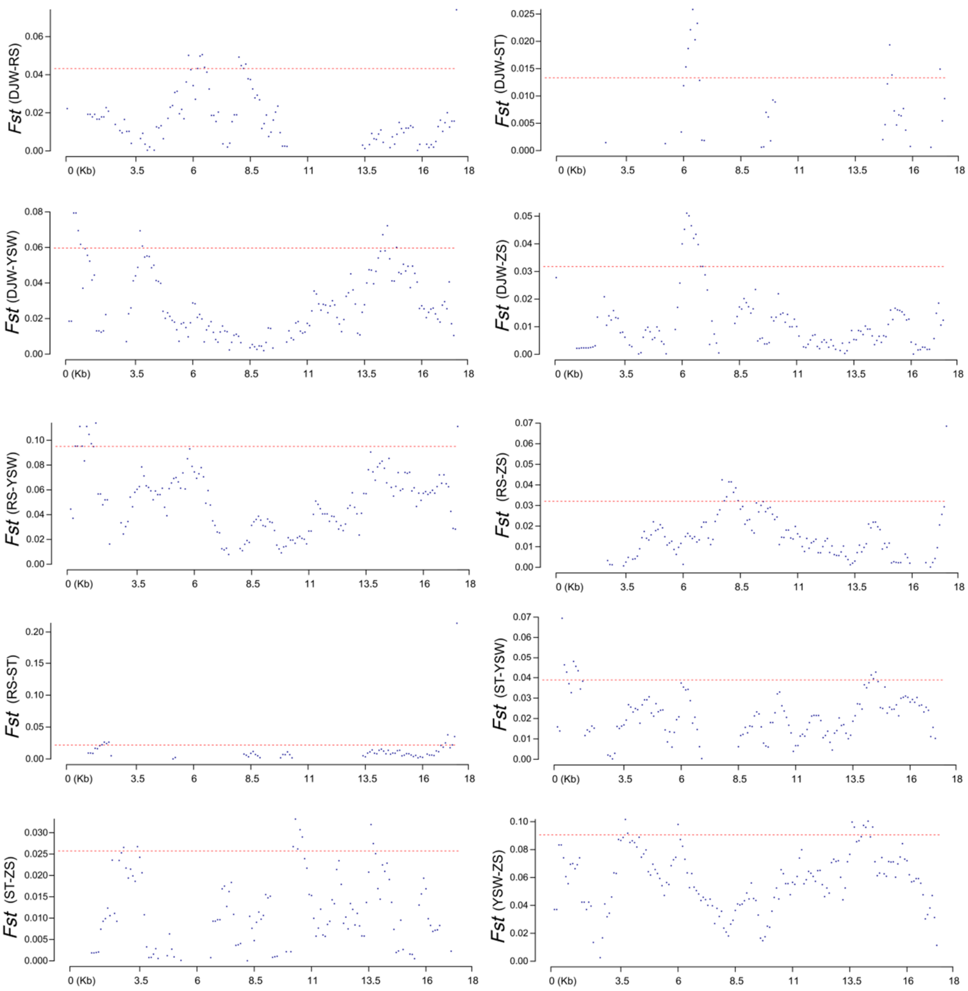

Figure S1. Population divergence (*Fst*) between the DJW, YSW, ZS, RS, and ST.

Supplementary Materials

The following are available online at https://www.mdpi.com/article/doi/s1 **Figure S1 :** Mitochondrial genome wide *Fst* distribution patterns reflecting genetic divergence among populations (DJW, YSW, ZS, RS, and ST). These dots present the distribution of *Fst* values across the mitochondrial genome, with the x - axis representing mitochondrial genomic physical distance (in Kb) and the y - axis indicating *Fst* values. They visualize the genetic differentiation levels between different population groups at the mitochondrial genomic level, aiding in the analysis of population genetic structure and potential selection signals.
